# Oxidative Stress and Inflammatory Biomarkers in Aqueous Humor and Blood of Patients with Leber’s Hereditary Optic Neuropathy

**DOI:** 10.3390/antiox15010051

**Published:** 2025-12-30

**Authors:** Berta Sánchez-Fernández, Pablo Zamorano-González, Elisa Martín-Montañez, Carmen Alba-Linero, Francisca Rius-Díaz, María García-Fernandez, Rafael Luque-Aranda, Ignacio García-Basterra

**Affiliations:** 1 Servicio de Oftalmología, Hospital Universitario Virgen de la Victoria, Campus de Teatinos, 29010 Málaga, Spain; carmen.alba.linero@uma.es (C.A.-L.); rafael.luque.aranda.sspa@juntadeandalucia.es (R.L.-A.); ignacio.garcia.basterra.sspa@juntadeandalucia.es (I.G.-B.); 2 Departamento de Farmacología y Pediatría, Facultad de Medicina, Instituto de Investigación Biomédica de Málaga y Plataforma en Nanomedicina-IBIMA Plataforma BIONAND, Universidad de Málaga, 29010 Málaga, Spain; 3 Grupo de Investigación Oftalmología, Instituto de Investigación Biomédica de Málaga y Plataforma en Nanomedicina-IBIMA Plataforma BIONAND, Universidad de Málaga, 29010 Málaga, Spain; 4 Departamento de Fisiología Humana, Facultad de Medicina, Instituto de Investigación Biomédica de Málaga y Plataforma en Nanomedicina-IBIMA Plataforma BIONAND, Universidad de Málaga, 29010 Málaga, Spain; pablozglez@uma.es (P.Z.-G.); igf@uma.es (M.G.-F.); 5 Departamento de Radiología y Medicina Física, Oftalmología y Otorrinolaringología, Facultad de Medicina, Universidad de Málaga, 29010 Málaga, Spain; 6 Departamento de Medicina Preventiva y Salud Pública, Facultad de Medicina, Universidad de Málaga, 29010 Málaga, Spain; rius@uma.es

**Keywords:** aqueous humor, serum, oxidative stress, inflammation, biomarkers, Leber’s Hereditary Optic Neuropathy

## Abstract

Leber’s hereditary optic neuropathy (LHON) is a mitochondrial disorder that causes visual impairment due to the degeneration of retinal ganglion cells. Oxidative stress (OS) and inflammatory cytokines have been implicated in its pathophysiology. We investigated, for the first time, the presence of OS biomarkers and inflammatory cytokines in the aqueous humor and peripheral blood of LHON patients compared to controls, aiming to identify potential clinical biomarkers for diagnosis and disease monitoring. A total of 38 participants were enrolled in a single-center, retrospective observational study, including 17 genetically confirmed LHON patients from different Spanish regions and 21 controls. OS biomarkers and inflammatory cytokines were quantified using spectrophotometry and fluorimetry techniques. Statistical analyses were performed to compare groups and to assess the discriminatory performance of biomarkers in identifying affected individuals. Compared to controls, LHON patients exhibited significantly higher levels of AOPP, LOOH, nitrotyrosine, GPX, GRD, and OX/AntiOX ratio in both aqueous humor and serum. Among these, serum LOOH levels and the OX/AntiOX ratio were the most reliable for identifying patients affected, with high sensitivity and specificity. However, additional data on serum IL-1ra are required to confirm its potential as an effective classifier. These findings highlight novel candidate biomarkers for the diagnosis and monitoring of LHON progression.

## 1. Introduction

Leber’s hereditary optic neuropathy (LHON) is a rare maternally inherited disease characterized by mitochondrial dysfunction and selective loss of retinal ganglion cells (RGCs) [1]. It constitutes the most prevalent optic neuropathy caused by a primary mutation in mitochondrial DNA (mtDNA) [2] and is the prevailing primary mtDNA disorder, with an estimated prevalence data ranging from 1/27,000 to 1/54,000 in Europe, but lower in Australia and Serbia (1/113,300 and 1/526,000, respectively) [3]. According to a regional study in Spain, the prevalence of LHON estimated was lower than Europe [4]. Symptoms entail painless visual loss with a subsequent central scotoma of acute or subacute onset that may affect both eyes, with or without a time interval between them. Typically, the disorder affects males between the ages of 15 and 35 years, representing a significant cause of legal blindness [1].

The physiopathology of LHON consists of a dysfunction in the electron transport chain, leading to a decreased energy synthesis and an increased production of reactive oxygen species (ROS) resulting in oxidative stress (OS) status [5,6]. OS is characterized by an imbalance where the generation of pro-oxidant agents surpasses the capacity of the antioxidant system to neutralize them. As observed for all three primary LHON mutations affecting mitochondrial complex I (3460G>A, 11778G>A, and 14484T>C), complex I–driven ATP synthesis is reduced. The excess production of ROS is of particular importance, whereas the decrease in ATP generation appears comparatively moderate [7]. Notably, primary LHON mutations differentially influence the underlying pathogenic mechanisms. For example, the 3460 mutation markedly slows electron transfer to coenzyme Q10, creating conditions that favor excessive ROS formation [5], whereas the 14,484 mutation is more closely associated with ATP depletion. Under these conditions, mitochondrial dysfunction ensues, ultimately resulting in the apoptosis of RGCs [8]. Due to their compact size and significant metabolic demands, RGC axons are particularly susceptible to OS [1,9,10]. Anatomically, the retinal nerve fiber layer (RNFL) is unmyelinated to preserve retinal optical transparency, a feature that increases its metabolic stress and vulnerability. This vulnerability is exacerbated in the smallest axons, particularly within the papillomacular bundle, where metabolic demand is concentrated and the fibers exhibit small diameters, reduced volume, and consequently fewer mitochondria. According to the Nerve Fiber Layer Stress Index equation—a mathematical model that predicts the order of fiber loss with high precision based on the ratio of metabolic demand to energy supply [11]—these axons suffer from adverse surface area to volume ratio. In this model, the former reflects the metabolic cost of repolarization, whereas the latter represents the mitochondrial content available to meet energy requirements.

Previous studies have demonstrated the involvement of OS as a key factor in LHON [12]: in animal models [13,14], in patient peripheral blood mononuclear cells [15], in patient-derived fibroblasts [16,17,18], and in blood samples [8,19]. To date, research on the presence of aqueous humor OS biomarkers has been limited to other pathologies like retinitis pigmentosa [20], glaucoma [21] age-related macular degeneration [22], or congenital hereditary endothelial dystrophy [23].

OS and inflammation are closely interconnected and can mutually reinforce each other [24]. Several inflammatory cytokines have been linked to neurodegenerative ocular diseases such as glaucoma [25,26] or macular degeneration [27]. Until now, this inflammatory imbalance has only been examined in LHON using patient-derived fibroblasts [28] and animal models [29].

The purpose of our study is, for the first time, to analyze the presence of OS and inflammatory cytokine biomarkers in both aqueous humor and peripheral blood of LHON patients, and to compare the results with those in control subjects, aiming to identify potential clinical biomarkers for diagnosis and disease monitoring. Additionally, we explored potentially influential factors in the measurements such as alcohol, tobacco, or idebenone consumption. We demonstrated the comparability between the markers analyzed in aqueous humor and serum, identifying the use of oxidative stress and inflammatory biomarkers as potential tools for early diagnosis and disease LHON monitoring. Particularly, the lipid hydroperoxides levels and redox balance, by correlating the oxidant/antioxidant ratio in serum, emerge as possible effective classifiers with high specificity, whereas conclusive future data on serum interleukin 1 receptor antagonist will be necessary to propose this biomarker as a potential effective classifier. Our findings provide a basis for the development of novel, non-invasive diagnostic tools and therapeutic monitoring strategies in individuals carrying LHON-associated mtDNA mutations (including asymptomatic carriers), as well as in patients with clinical suspicion of LHON while awaiting genetic confirmation.

## 2. Materials and Methods

### 2.1. Study Design and Participants

This was a single-center, retrospective observational study in which a total of 38 patients donated aqueous humor and blood. Seventeen patients diagnosed with LHON from different Spanish regions were enrolled in the study. All of them had both clinical and genetic confirmation of the disease. Twenty-one patients undergoing cataract surgery free from other ophthalmological or systemic pathological conditions acted as controls. Asymptomatic LHON mutation carriers were not enrolled in this study. Patients under 18 years and pregnant women were excluded. In LHON patients, the eye with the worst visual acuity was selected for sampling. The sample size was calculated using the following formula: adjusted sample = n/(1 − R). Considering that 16 subjects without losses were estimated in each group (n), with an expected proportion of losses (R) set at 0.05, a minimum of 17 subjects should be included in each study group.

Patients diagnosed with chronic-stage LHON were recruited from different hospitals throughout the country, with many of them referred through the National Leber Optic Nerve Atrophy Association (ASANOL). All the controls came from the Ophthalmology Department of Virgen de la Victoria University Hospital (Malaga, Spain). All samples were collected exclusively at Virgen de la Victoria University Hospital by the same research team. The Ethics Committee of Virgen de la Victoria University Hospital of Malaga, Spain, approved this study on 25 August 2022 (code 0392-N-21), in accordance with the Declaration of Helsinki. Written informed consent was obtained from all participants. The study was conducted blindly. Neither the researchers who analyzed the data nor those who examined the samples were aware of the group each sample belonged to.

All the patients filled out a comprehensive anamnesis questionnaire considering information on factors such as smoking status, alcohol consumption, and the use of the potent intramitochondrial antioxidant idebenone. We calculated cigarette smoking with the pack-years formula [30]. To assess alcohol consumption, we employed standard drinking units (SDUs) as defined by the Spanish Scientific Society for Research on Alcohol, Alcoholism, and Other Drug Addictions as 1 SDU equivalent to 10 g of pure alcohol [31].

### 2.2. Ophthalmic Examination

A comprehensive ophthalmic examination, including measurement of best-corrected visual acuity (BCVA), intraocular pressure (IOP), and axial length (AL), followed by an optical coherence tomography (OCT), was performed in all the patients. The BCVA was quantified by Snellen visual acuity charts. For patients who were unable to read the chart, a scale of “counting fingers”, “hand movements”, and “light or no light perception” was used. AL was measured using IOLMaster^®^ 500 (Carl Zeiss, Berlin, Germany). Retinal nerve fiber layer (RNFL) and RGC layer (RGCL) were studied using swept-source (SS)-OCT (Triton^®^, Tokyo, Japan) with a 12 × 9 mm wide protocol scan.

### 2.3. Samples Collection

The aqueous humor samples (100–200 µL) were collected through anterior chamber paracentesis in the operating room under sterile conditions. A periorbital skin scrubbing with 10% povidone-iodine (PVI) and a drop of 5% PVI were both applied for a minimum of three minutes before ocular extraction. Aqueous humor samples from 17 LHON patients were obtained using a 30-gauge needle on a tuberculin syringe (1 mL) via paracentesis under a microscope. We also collected aqueous humor samples from 21 disease-free patients scheduled for cataract surgery, aspirating through a routine paracentesis with a 25-gauge anterior chamber cannula on a tuberculin syringe (1 mL) as the first step of the cataract surgery. All the undiluted samples were placed in sterile tubes. They were immediately deposited in liquid nitrogen and stored at −80 °C until use.

Blood samples from the 38 fasting participants were collected into 4 mL EDTA clot activator tubes. Samples were centrifuged at 2500 rpm for 10 min, and later supernatants were divided into aliquots, which were frozen at −80 °C until biochemical determinations.

### 2.4. Measurement of Oxidative Stress and Inflammatory Cytokine Biomarkers

Advanced oxidation protein products (AOPPs) and lipid hydroperoxides (LOOH) were measured. AOPP levels were measured using a microassay adapted to Cobas Mira following the methodology of Matteuci et al. [32] founded on the original approach by Witko-Sarsat et al. [33]. LOOH analysis was performed using the FOX2 method (Ferrous Oxidation). The automated analyzer Cobas Mira (Roche Diagnostics, Barcelona, Sapin) was used to measure the following OS markers by spectrophotometry, automated by Arab and Steghens [34] and tailored for Cobas Mira at a wavelength of 600 nm.

Nitrotyrosine levels were determined by employing a commercially available enzyme-linked immunosorbent assay (ELISA) kit (ab210603, Abcam, Amsterdam, The Netherlands) following the manufacturer’s protocol. Aqueous humor levels were expressed as nmol/mg and serum levels as µM.

The ProcartaPlex multiplex Immunoassay (Thermo Fisher Scientific, Waltham, MA, USA), a bead-based protein quantification assay that follows the principles of a sandwich ELISA, was used to measure the following inflammatory markers: Interleukin-1 β (IL-1 β), IL-6, IL-12p40, leptin, interferon- γ (IFN-γ), and tumor necrosis factor α (TNF-α). Biomarker levels were determined following the manufacturer’s instructions. Aqueous humor and serum levels were expressed as pg/mL. A description of these biomarkers and their clinical relevance can be found in Supplementary Table S1.

### 2.5. Measurement of Antioxidants and Anti-Inflammatory Cytokine Biomarkers

The automated analyzer Cobas Mira (Roche Diagnostics, Barcelona, Sapin) was used to measure the following antioxidants markers using Randox commercial kits (Randox Laboratories Ltd., Barcelona, Spain) according to the manufacturer’s instructions: total antioxidant status (TAS) using the Randox NX2332 kit, glutathione peroxidase (GPx) using the Randox RS504 kit, glutathione reductase (GRD) using the Randox GR2368 kit, and superoxide dismutase (SOD) using the Randox SD125 kit. Thus, aqueous humor levels were expressed as µmol/mg (TAS) and U/mg (GPX. GRD) and serum levels as mM (TAS), U/L (GPX, GRD) and mU/ng (SOD). Glucose 6 Phosphate Dehydrogenase (G6PDH) was quantified by using a Biochemichal Enterprise kit (BEN s.r.l G6P8905, Milan, Italy) in the same automated analyzer following the product instructions. Aqueous humor G6PDH levels were expressed as mU/mg and serum levels as U/L. The extracellular superoxide dismutase (SOD3) levels were measured in both serum and aqueous humor samples using an ELISA kit (Cusabio CSB-EL022399HU, Madrid, Spain), according to the manufacturer’s instructions. Aqueous humor levels were expressed as pg/mg and serum levels as ng/mL.

Cyclic guanosine monophosphate (cGMP), a nitric oxide downstream messenger, was measured by using an ELISA kit (ab133052, Abcam, Amsterdam, The Netherlands) following the manufacturer’s guidelines. cGMP serum levels were expressed as U/mL.

The ProcartaPlex multiplex Immunoassay (Thermo Fisher Scientific, Waltham, MA, USA), a bead-based protein quantification assay that follows the principles of a sandwich ELISA, was used to measure the following anti-inflammatory markers: interleukin 1 receptor antagonist (IL-1ra), IL-4, IL-10, and IL-13. These cytokine levels were determined following the manufacturer’s instructions. Aqueous humor and serum levels were expressed as pg/mL (see Supplementary Table S1 for biomarker descriptions).

### 2.6. Statistical Analyses

The t-test and the Mann–Whitney U test were used to compare the difference between two continuous variables based on whether they followed a normal distribution or not, while also considering the size of the analyzed sample. The Shapiro–Wilk test was applied to evaluate whether continuous variables followed a normal distribution. For variables demonstrating a normal distribution, Levene’s test was subsequently applied to ensure the homogeneity of variances before the t-test. For qualitative variables, the Chi-square test was performed.

Chi-squared Automatic Interaction Detection (CHAID) decision trees were applied to significant biomarkers from univariate analysis to determine their optimal cut-off points for distinguishing between LHON patients and healthy controls. Based on these predefined cut-off points, the cumulative number of elevated risk biomarkers was calculated for each participant. Cross-tabulations were performed to examine the relationship between the number of risk biomarkers and LHON diagnosis, considering patients with complete datasets (n = 11). An exploratory three-factor factorial analysis of variance (ANOVA) (LHON status, smoking, and alcohol consumption) was performed to explore potential cofounding factors and their interactions. Significance was determined at a threshold of 0.05.

The SPSS^®^ software for Windows^®^ version 11.0 (SPSS Inc., Chicago, IL, USA) was used to perform all statistical analyses. An online tool [35] was employed for outlier detection and management within the dataset.

## 3. Results

### 3.1. Participants Characteristics

In total, 38 participants were included in the work. Of these, 17 were diagnosed with LHON, and 21 were undergoing cataract surgery free from other ophthalmological or systemic pathological conditions, serving as the control group. The demographic, epidemiological, and clinical details of the subjects are shown in Table 1. The groups demonstrated similar demographic and smoking and alcohol status and comparable clinical profiles, differing only in disease-specific characteristics. As expected, BCVA was significantly higher in the control group (0.10 ± 0.24) compared with LHON patients (0.96 ± 0.21, *p* < 0.05), as well as the average thickness of RNFL (95.23 ± 4.08) and RGCL (68.15 ± 2.28, which showed lower values in the LHON group (54.65 ± 23.12 and 39.35 ± 6.08, respectively, *p* < 0.05).

Among LHON patients, all of them with clinical and genetic confirmation of the disease, six subjects (35.3%) presented the primary mutation 11778G>A, while another six (35.3%) were identified with the 3460G>A. No cases of the 14484T>C mutation were detected. Secondary mutations were observed in two patients (11.76%) with the 3734A>G variant. Additionally, three participants presented combined mutations: one with 11778 G>A + 13708G>A (5.88%) and two with 4216T>C + 13708G>A (11.76%).

### 3.2. Oxidative Stress Biomarkers and Cytokines Analysis in Aqueous Humor

Oxidant and antioxidants biomarker measures are presented in Table 2. Concerning oxidant biomarker levels, all showed statistically significant elevation in the aqueous humor of LHON patients (AOPP, *p* = 0.046; LOOH, *p* = 0.023; and nitrotyrosine, *p* = 0.048). About antioxidants, SOD3 levels were higher in controls (*p* = 0.040), whereas GPx and GRD levels showed a significant increase in LHON patients (*p* = 0.006 and *p* = 0.048, respectively). These results showed differences in oxidant-antioxidant status between the two groups.

The capacity of the body to withstand oxidative damage relies on the efficiency of its antioxidant defenses to counteract oxidative damage. Therefore, the redox balance by correlating the oxidant/antioxidant ratio (OX/AntiOX ratio) was analyzed [36]. Thus, the ratio between parameters of oxidative damage and antioxidant parameters significantly increased in LHON patients (0.82 ± 0.58) compared with controls (0.45 ± 0.17, *p* = 0.021) suggesting a more oxidant state than in controls.

Among the cytokines examined, only two biomarkers showed statistical differences between the two groups: the pro-inflammatory marker leptin and the anti-inflammatory marker IL-1ra (Table 2). Leptin levels were higher in the control group (340.85 ± 239.55 vs. 190.67 ± 58.59, *p* = 0.047) and IL-1ra levels in the LHON group (499.67 ± 161.73 vs. 302.72 ± 89.17, *p* = 0.007).

### 3.3. Oxidative Stress Biomarkers and Cytokines Analysis in Blood

To ensure potential outcomes and comparability, blood samples underwent the same measurements, additionally including the assessment of SOD enzymatic activity and cGMP levels. Table 3 presents descriptive statistics for each parameter analyzed.

The results concerning oxidant biomarkers were consistent with those obtained in aqueous humor, showing a significant increase in LOOH and nitrotyrosine in the LHON group (LOOH, *p* = 0.05; nitrotyrosine, *p* = 0.04) and a tendency of AOPP to increase in LHON patients (*p* = 0.053). In relation to antioxidants, GRD levels and SOD activity were significantly higher in the LHON group (*p* = 0.035, and *p* = 0.036, respectively). The OX/antiOX ratio significantly increased in LHON patients (13.17 ± 3.95) compared with controls (10.35 ± 1.58, *p* = 0.021), suggesting again a more oxidant state than in controls.

In terms of the cytokine profile of the two groups, IL-1β, TNF-α, and IL-1ra were found to be elevated in the control group (IL-1β, *p* = 0.004; TNF- α, *p* = 0.009; IL-1ra, *p* = 0.015). This difference in cytokine profiles between aqueous humor and blood should be mainly due to the blood–aqueous barrier, allowing the aqueous humor to have its own cytokine profile.

### 3.4. Analysis in LHON Subgroups

Due to the reported association between tobacco and alcohol with the disease development [6], along with the protective effect of idebenone [37], a synthetic CoQ derivative with a potent intramitochondrial antioxidant effect [38], an explorative analysis was performed in the LHON group. The Mann–Whitney U test was used to investigate the previously mentioned biomarkers and variables in patients exposed to tobacco, alcohol, and idebenone consumption. The statistically significant analysis is shown in Table 4.

Aside from the clinical differences detected in layer thicknesses after alcohol and tobacco consumption, few differences are observed in the biomarker profile. Thus, among LHON patients, smoking was associated with reduced RGC average thickness (*p* = 0.002), a measure of optic nerve damage, as well as lower aqueous humor levels of anti-inflammatory cytokines IL-13 and IL-1ra compared to non-smokers (*p* = 0.009). Concerning LHON patients who consumed alcohol, statistically significant differences were found, as they revealed a reduced RNFL average thickness (*p* = 0.027) and lower levels of the plasmatic pro-inflammatory factor TNF-α (*p* = 0.014). Finally, patients receiving idebenone showed lower levels of GPX in aqueous humor (*p* = 0.018).

### 3.5. Adjustment Analysis for Potential Confounders: Smoking and Alcohol

In order to analyze potential confounders, we performed an exploratory adjusted analysis using factorial ANOVA, including LHON status, smoking, and alcohol consumption for those outcomes, showing significant group differences from Table 4. The significance of the main LHON effect remained consistent with those reported in Table 1, Table 2 and Table 3, suggesting that these differences were not explained by smoking or alcohol consumption in our cohort.

### 3.6. Statistical Exploration of Biomarkers for LHON Risk Assessment

An explorative and comparative analysis of biomarkers for LHON risk assessment was developed. Table 5 shows the statistically significant analysis.

Thus, IL-1ra in aqueous humor demonstrated the highest overall classification rate (OCR = 85%) and area under curve (AUC = 0.869). Nevertheless, the biomarkers showed as optimal classifiers were LOOH and OX/antiOX ratio, both in serum, given their notable OCR (78.8% and 76.9%, respectively) and AUC (0.807 and 0.776, respectively) supported by their strong performance in sensitivity (73.3%) and specificity (83.3% and 81.8%, respectively) measurements. Both biomarkers were significantly elevated in the LHON group, with cut-off points of 19.0 µM (LOOH) and 11.19 µM (OX/antiOX). These serum biomarkers could enable the accurate differentiation of patients with and without the disease.

### 3.7. Cumulative Risk Analysis

To evaluate the relationship between the “number of elevated risk parameters and the probability of having LHON”, we created a variable termed Number of High-Risk Parameters. This new variable represents the sum of the risk parameters presented in each patient, based on the predefined cut-off points obtained in CHAID. Cross-tabulations were performed to examine its relationship with LHON diagnosis, considering only patients with complete data analyzed (n = 11).

Our results indicate that having two or more elevated risk parameters considerably increases the probability of LHON diagnosis. All patients with LHON (100%) presented at least two risk parameters, whereas none (0%) of the controls showed ≥ 2 risk parameters.

## 4. Discussion

Previous studies have demonstrated a link between OS and the etiology and pathophysiology of LHON [13,14,15,16,17,18], although only a limited number of investigations have specifically evaluated the role of OS markers in serum samples from LHON patients [8]. OS and inflammation are closely interconnected and can mutually reinforce each other [24]. Some studies have demonstrated that inflammatory response plays a role in neurodegenerative ocular diseases [25,26,27]. A possible interpretation in the context of LHON is that inflammatory response may arise as a consequence of mitochondrial dysfunction. However, studies on the involvement of the inflammatory response in the pathophysiology of LHON are limited and focused on in vitro or animal models [28,29], without conclusive data that rely on clinical samples.

To our knowledge, our study is the first research to report OS and inflammatory cytokine biomarker levels in both aqueous humor and peripheral blood of patients with LHON. We measured oxidant/antioxidant markers and pro-inflammatory/anti-inflammatory cytokines and compared the results with those in control subjects, aiming to identify potential clinical biomarkers for diagnosis and disease monitoring.

Concerning OS profile, our data indicated significantly higher aqueous humor levels in all of the oxidant markers measured in LHON patients as compared to controls, and similar results were found in serum samples. Thus, AOPP, LOOH, and nitrotyrosine levels were increased in aqueous humor, and LOOH and nitrotyrosine, in the case of serum, showed a trend toward an increase in AOPP levels. These data are consistent with the previous study of Rovcanin et al. [8], where the overall extent of oxidation was determined by assaying total oxidant status (TOS) in plasma samples of LHON patients. They found significantly increased TOS and AOPP levels. This is also consistent with the case report published by Falabella et al. [15] about the cross interactions between reactive ROS and reactive nitrogen species (RNS) in peripheral blood mononuclear cells derived from a female patient. This study showed increased levels of both ROS and RNS. The interplay between these stress pathways seems to contribute to the mitochondrial dysfunction presented in LHON, damaging RGCs.

In relation to antioxidant markers, the levels of the key enzymes involved in the glutathione redox cycle, GPX and GRD, were significantly elevated in aqueous humor in LHON patients. The patients receiving idebenone showed significantly lower levels of GPX than those found in untreated patients but higher than controls. Aqueous humor data are consistent with those found in serum samples, where significantly higher GRD levels and a tendency of GPX levels to increase in LHON patients were observed. The SOD measures in both types of samples were also different in LHON patients than those found in the control group. The profile of antioxidative enzymes found in our study were related to the findings of the study developed in cybrid cells carrying the most common LHON mutations [17]. SOD, GPx, and GR activities were altered in cells carrying the 11778G>A and 3460G>A mutations compared with controls. These mutations, in which excess ROS production is particularly relevant, were the most frequent LHON variants in our cohort, whereas we observed no cases of the 14484T>C mutation.

The capacity of the body to withstand oxidative damage relies on the efficiency of its antioxidant defenses to counteract oxidative damage. Therefore, the OX/AntiOX ratio analysis is considered a good marker to evaluate the redox balance [36]. Our data indicate that in both aqueous humor and serum, the ratio between parameters of oxidative damage and antioxidant parameters significantly increased in LHON patients compared with controls, suggesting a more oxidant state in LHON patients than in control subjects, which definitely implies the presence of redox imbalance. Our data observed in serum confirm the previously described findings in plasma samples of LHON patients [8], where high oxidative stress index (defined as the ratio of TOS to TAS parameters) was found in LHON patients compared to the control group.

Knowledge of all these findings show that the presence of the redox imbalance represents an important pathophysiological event in LHON and that OS markers could potentially serve as biomarkers for diagnosis and disease monitoring. In this sense, we provide statistical evidence reinforcing the involvement of OS as a key factor in the pathogenesis of LHON, with a focus on its clinical application by means of the development of an explorative and comparative analysis of biomarkers for LHON risk assessment. Among the biomarkers we evaluated, LOOH and the OX/AntiOX ratio in serum emerged as the most effective classifiers, presenting significant OCR and AUC values, along with high sensitivity and specificity assessments. Both biomarkers were significantly elevated in the LHON group, with cut-off points of 19.0 µM (LOOH) and 11.19 µM (OX/antiOX). Our results in aqueous humor and serum are comparable and therefore ensure reliable outcomes. These serum biomarkers could enable the accurate differentiation of patients with affected and without clinical evidence of LHON (carriers) and with and without treatment. They offer a less invasive and more accessible option for routine patient monitoring compared to the difficulties of aqueous humor sample collection, while also demonstrating significant clinical utility due to their high specificity. This is important in an uncommon disease such as LHON, where the distinction between asymptomatic carriers and those at risk of developing OS-related pathology may be particularly relevant. Normal LOOH level and OX/antiOX ratio suggest a healthy oxidative status, which may reduce the likelihood of progressing to a clinical diagnosis of LHON while awaiting genetic testing results. Values below their risk cut-off points would support a healthy and balanced redox state, which could be helpful in managing asymptomatic carriers or relatives of patients with LHON, facilitating the study of disease progression over time. Future studies could evaluate whether redox biomarkers can help monitor response to idebenone or other emerging therapies. Any potential role in individualized dosing would require prospective validation.

In the context of the oxidative damage of lipids in the eye’s membranes, which is implicated in eye damage, lipid peroxidation contributes to the progression of eye diseases [39]. Thus, the role of anti-lipid peroxidation tools against RGC damage and optic nerve degeneration is being studied after traumatic and glaucomatous conditions in animals [40]. Furthermore, idebenone, the only disease-specific drug approved to treat visual impairment in LHON patients, inhibited mitochondrial lipid peroxidation [41]. Our findings on serum LOOH levels, which represent one of the most effective classifier biomarkers, along with the described role of lipid peroxidation, support the use of LOOH as a fast and cost-effective clinical tool for patient stratification.

Concerning the inflammatory profile, we observed differences between aqueous humor and serum, probably due to the blood–aqueous barrier functions and the disease stage of the recruited LHON patients, who were all in the chronic phase (>1 year after onset). Our data indicated that the concentrations of inflammatory cytokines were not significantly different between LHON patients and control subjects in aqueous humor, except for leptin and IL-1ra levels. We observed lower leptin levels in LHON patients than those found in the control group. This result is in agreement with those recently reported by Candan and Firat [42] about the evaluation of ocular structures in patients with congenital leptin deficiency, where it is suggested that the lack or low levels of leptin may contribute to optical decline. Moreover, evidence supports the presence of leptin receptors in the eye, including within the RGCL [43]. The higher IL-1ra levels found in the LHON group may indicate a compensatory response mechanism to counteract inflammation and preserve ocular homeostasis [44].

Regarding inflammatory serum markers, IL-1β, TNF- α, and IL-1ra were significantly elevated in the control subjects. As mentioned before, we recruited disease-free patients scheduled for cataract surgery as controls, with the purpose of analyzing the presence of OS and inflammatory cytokine biomarkers in both aqueous humor and peripheral blood of LHON patients, and comparing the results with those of the control subjects. The inflammatory profile found in our control subjects (cataract patients) is consistent with the findings of the causal associations study between circulating inflammatory cytokines and eye disease carried out by Teng et al. [27], where IL-1ra was potentially associated with the increased risk of cataract. The IL-1ra upregulation observed may be related to the compensatory response to inflammation caused by the high IL-1β and TNF- α levels found in our work [44,45].

Taking inflammatory cytokine findings into account, we developed the explorative and comparative analysis of these biomarkers for LHON risk assessment. Thus, IL-1ra demonstrated the highest OCR and AUC in aqueous humor and a high elevated OCR in serum with a low AUC, probably due to the specific characteristic of our control subjects. Additional studies including cataract-free control subjects are necessary to propose IL-1ra as a serum biomarker capable of accurately differentiating patients with and without the disease.

Additionally, it is well established that the primary mtDNA mutations is necessary but not sufficient for the development of neuropathy, and lifestyle factors such as smoking and alcohol consumption have been shown to act as frequent disease triggers [6,46,47]. Tobacco smoking is the most common, with or without concurrent alcohol intake, and the pattern of consumption of these factors is relevant to their association with loss of vision. Thus, maximum-intensity drinking is probably related to visual loss [47], as are cumulative smoking and maximum-intensity smoking [46,47]. However, chronic smoking could exert both protective and detrimental effects, which may explain why both Kirkman et al. [47] and Carelli et al. [46] reported paradoxical results at different levels of smoking. They found that patients with the heaviest smoking histories experienced the latest onset of the disease, whereas light smoking slightly accelerated the rate of visual loss compared with non-smokers. In our study, adjustment for potential confounders smoking and alcohol consumption did not change the main LHON effect observed, suggesting that our results were not explained by smoking or alcohol consumption.

From a clinical perspective, our findings point to the critical role of monitoring OS and inflammatory biomarkers in tracking LHON progression. The identification of specific biomarkers, which are elevated in LHON, offers a pathway for early diagnosis and timely intervention, potentially delaying or preventing the onset of visual symptoms in asymptomatic carriers. This understanding is consistent with the increasing awareness that genetic predisposition and environmental factors are crucial in the phenotypic expression of LHON, emphasizing the need for developing personalized treatment strategies tailored to individual risk profiles [48].

Although our findings provide valuable insights into the OS and inflammatory profiles in LHON, this study is not without its limitations. The small sample size due to LHON being a rare disease, though sufficient for our exploratory observations, may not fully represent the broader spectrum of the disease, and the cross-sectional design reflects a single point in disease progression. Secondly, we were unable to recruit asymptomatic mutation carriers willing to undergo aqueous humor sampling. Third, the small numbers within each subgroup meant that we could not reliably analyze results by specific mtDNA mutations or by detailed smoking or alcohol-consumption patterns. Therefore, subgroup analyses were considered exploratory. Future studies in larger and more diverse cohorts are essential to validate these biomarkers and examine their potential for long-term prediction and monitoring disease progression.

## 5. Conclusions

The present study provides support for the potential link between OS and inflammation with the pathophysiology of LHON. The analysis of the following OS biomarkers AOPP, LOOH, nitrotyrosine, GPX, GRD, and SOD, along with the OX/AntiOX ratio and the cytokine IL-1ra, in both aqueous humor and serum of LHON patients, suggest their potential use as tools for early diagnosis and disease monitoring. Particularly, the fast and cost-effective biomarker LOOH and the redox balance by correlating the OX/AntiOX ratio in serum, emerge as possible effective classifiers with high specificity, supporting a healthy oxidative status in individuals whose levels fall below the risk cut-off point. However, conclusive future data on serum cytokine IL-1ra will be necessary to propose this biomarker as a potential effective classifier. Our results provide a basis for the development of novel, non-invasive diagnostic tools and therapeutic monitoring of LHON, focused in preventing or delaying vision loss in genetically predisposed individuals.

## Figures and Tables

**Table 1:** Demographic, epidemiological, and clinical profile of participants.

Variables	Control (n = 21)	LHON (n = 17)
Age (years)	54.29 ± 15.35	45.71 ± 15.57
Gender (male) Gender (female)	10/21 (47.6%) 11/21 (53.4%)	11/17 (64.7%) 6/17 (35.3%)
Caucasian (yes) Caucasian (no)	21/21 (100%) 0/21 (0%)	15/17 (88.23%) 2/17 (11.76%)
Smoking (packs/years)	8.81 ± 23.32	12.76 ± 26.87
Alcohol (SDU)	3.42 ± 6.76	8.23 ± 8.64
Idebenone (yes) Idebenone (no)	0/21 (0%) 21/21 (100%)	11/17 (64.71%) 6/17 (35.29%)
BCVA *	0.96 ± 0.21	0.10 ± 0.24
AL (mm)	23.85 ± 0.55	23.55 ± 1.17
IOP (mmHg)	15.85 ± 0.88	16.70 ± 2.11
RNFL (µ) *	95.23 ± 4.08	54.65 ± 23.12
RGCL (µ) *	68.15 ± 2.28	39.35 ± 6.08

LHON patients and free-disease patients (control) showed similar demographic, epidemiological, and clinical profile, with differences limited to optic neuropathy-specific features. SDU—standard drinking units; BCVA—best-corrected visual acuity; AL—axial length; IOP—intraocular pressure; RNFL—retinal nerve fiber layer; RGCL—retinal ganglion cell layer. Values are expressed as number (%) or mean ± standard deviation (SD). Student’s t, Mann–Whitney U, and Chi-square tests were conducted to analyze these differences between groups. * Statistically significant at *p* value <0.05.

**Table 2:** Aqueous humor oxidative stress and inflammation biomarkers.

Markers		Control		LHON
Oxidant-Antioxidants	n	Mean ± SD	n	Mean ± SD
AOPP *	19	77.79 ± 30.24	16	97.56 ± 25.27
LOOH *	19	183.86 ± 114.46	15	264.03 ± 80.85
Nitrotyrosine *	20	0.55 ± 0.47	17	1.66 ± 2.10
TAS	20	3.54 ± 3.01	16	2.85 ± 2.04
G6PDH	15	20.32 ± 17.24	11	29.70 ± 35.54
GPx *	17	0.91 ± 0.42	15	1.26 ± 0.22
GRD *	21	100.95 ± 43.72	16	110.51 ± 36.80
SOD3 *	19	195.98 ± 71.49	16	157.92 ± 23.36
Cytokines	n	Mean ± SD	n	Mean ± SD
IL-1 β	13	7.30 ± 2.52	12	7.09 ± 2.70
IL-6	12	69.33 ± 20.70	12	77.33 ± 32.58
IL-12p40	13	5.27 ± 1.77	12	5.14 ± 1.80
Leptin *	13	340.85 ± 239.55	12	190.67 ± 58.59
IFN- γ	13	30.24 ± 10.01	12	28.91 ± 10.50
TNF-α	13	18.64 ± 5.87	12	17.55 ± 5.61
IL-1ra *	11	302.72 ± 89.17	9	499.67 ± 161.73
IL-4	13	48.92 ± 15.90	12	47.33 ± 16.46
IL-10	13	7.33 ± 2.46	12	7.09 ± 2.57
IL-13	13	9.08 ± 2.98	12	8.37 ± 3.03

LHON patients and free-disease patients (control) showed differences in antioxidant-oxidants status and cytokine levels in aqueous humor. AOPP—Advanced oxidation protein products; LOOH—lipid hydroperoxides; TAS—total antioxidant status; G6PDH—Glucose 6 Phosphate Dehydrogenase; GPx—glutathione peroxidase; GRD—glutathione reductase; SOD3—extracellular superoxide dismutase; IL—interleukin; IFN- γ—interferon- γ; TNF-α—tumor necrosis factor α; IL-1ra—interleukin 1 receptor antagonist. * Statistically significant at *p* value <0.05.

**Table 3:** Blood oxidative stress and inflammation biomarkers.

Markers		Control		LHON
Oxidant-Antioxidants	n	Mean ± SD	n	Mean ± SD
AOPP	17	69.53 ± 10.67	15	84.07 ± 25.30
LOOH *	18	16.33 ± 3.85	15	24.87 ± 9.67
Nitrotyrosine *	15	176.64 ± 121.75	17	351.53 ± 188.14
TAS	19	1.44 ± 0.11	15	1.49 ± 0.10
G6PDH	17	63.34 ± 31.08	14	53.72 ± 21.94
GPx	19	786.4 ± 186.71	15	837.13 ± 130.35
GRD *	21	80.95 ± 10.90	17	90.23 ± 14.21
SOD3	18	364.78 ± 201.99	15	215.66 ± 197.81
SOD *	16	24.24 ± 15.33	12	51.91 ± 38.92
cGMP	20	0.24 ± 0.10	17	0.31 ± 0.25
Cytokines	n	Mean ± SD	n	Mean ± SD
IL-1 β *	19	4.75 ± 0.24	17	4.55 ± 0.12
IL-6	18	41.11 ± 1.28	14	40.26 ± 1.31
IL-12p40	19	3.60 ± 0.16	16	3.54 ± 0.86
Leptin	18	5691.11 ± 3566.22	16	4092.19 ± 2384.66
IFN- γ	17	21.25 ± 1.45	16	28.91 ± 10.50
TNF-α *	18	12.70 ± 0.66	15	12.08 ± 0.62
IL-1ra *	11	40.81 ± 21.07	14	21.50 ± 18.99
IL-4	19	32.97 ± 4.27	17	33.09 ± 7.07
IL-10	19	4.80 ± 0.24	17	4.91 ± 0.39
IL-13	19	5.67 ± 0.61	16	5.50 ± 0.14

LHON patients and free-disease patients (control) showed differences in antioxidant-oxidants status and cytokine levels in blood. AOPP—advanced oxidation protein products; LOOH—lipid hydroperoxides; TAS—total antioxidant status; G6PDH—Glucose 6 Phosphate Dehydrogenase; GPx—glutathione peroxidase; GRD—glutathione reductase; SOD3—extracellular superoxide dismutase; SOD—superoxide dismutase; cGMP—cyclic guanosine monophosphate; IL—interleukin; IFN- γ—interferon- γ; TNF-α—tumor necrosis factor α; IL-1ra—interleukin 1 receptor antagonist. * Statistically significant at *p* value <0.05.

**Table 4:** Explorative analysis of LHON subgroups based on risk and protective factors.

**Variables**		**Smoking**		**No Smoking**
	n	Mean ± SD	n	Mean ± SD
RGCL (µ) *	4	31.75 ± 5.31	13	41.69 ± 4.13
IL-13 (aqueous humor) *	3	5.40 ± 0.00	9	9.36 ± 2.88
IL-1ra (aqueous humor) *	3	400.67 ± 161.57	6	549.17 ± 150.30
**Variables**		**Alcohol**		**No Alcohol**
	n	Mean ± SD	n	Mean ± SD
RNFL (µ) *	9	42.56 ± 19.79	8	68.25 ± 19.37
TNF-α (serum) *	8	11.71 ± 0.38	7	12.51 ± 0.56
**Variable**		**Idebenone**		**No Idebenone**
GPx (aqueous humor) *	11	1.19 ± 0.22	4	1.45 ± 0.29

LHON patients showed clinical and oxidative/inflammatory status differences in the presence of risk and protective factors. RGCL—retinal ganglion cell layer; IL—interleukin; IL-1ra—interleukin 1 receptor antagonist; RNFL—retinal nerve fiber layer; TNF-α—tumor necrosis factor α; GPx—glutathione peroxidase. * Statistically significant at *p* value <0.05.

**Table 5:** Explorative and comparative analysis of biomarkers for LHON risk assessment.

**Biomarkers ** **(Aqueous Humor)**	**Cut-Off Point**	**LHON Below/Above Risk Value**	**Chi2 Significance**	**Odds Ratio**	**Sensitivity**	**Specificity**	**OCR**	**AUC**
OX/antiOX Ratio	≤0.730 >0.730	34.6% (9) 100% (6)	0.004	-	40%	100%	71.9%	0.696
GPX	≤1.066 >1.066	15.4% (2) 68.4% (13)	0.003	11.97	86.7%	64.7%	75%	0.769
Leptin	>230 ≤230	16.7% (2) 76.9% (10)	0.003	16.68	83.3%	76.%	80%	0.183
IL-1ra	≤470 >470	21.4% (3) 100% (6)	0.001	-	66.7%	100%	85%	0.869
**Biomarkers ** **(Blood)**	**Cut-Off Point**	**LHON Below/Above Risk Value**	**Chi2 Significance**	**Odds Ratio**	**Sensitivity**	**Specificity**	**OCR**	**AUC**
LOOH	≤19 >19	21.1% (4) 78.6% (11)	0.001	13.75	73.3%	83.3%	78.8%	0.807
OX/antiOX Ratio	≤11.19 >11.19	30.8% (4) 84.6% (11)	0.005	12.38	73.3%	81.8%	76.9%	0.776
SOD activity	≤53.82 >53.82	30.4% (7) 100% (5)	0.004	-	41.7%	100%	75%	0.724
IL-1ra	>19.32 ≤19.32	20% (3) 78.6% (11)	0.002	14.67	78.6%	80%	79.3%	0.207

Parameters related to the risk of having the disease. OCR—overall classification rate; AUC—area under curve; OX/antiOX ratio—oxidant/antioxidant ratio; GPx—glutathione peroxidase; IL-1ra—interleukin 1 receptor antagonist; SOD—superoxide dismutase. In certain parameters, the odds ratio cannot be computed due to the existence of cells with a frequency of zero.

## Data Availability

The original contributions presented in this study are included in the article/Supplementary Materials. Further inquiries can be directed to the corresponding author.

## Supplementary Materials

The following supporting information can be downloaded at https://www.mdpi.com/article/10.3390/antiox15010051/s1. Table S1: Oxidative stress and inflammatory biomarkers.

## Abbreviations

The following abbreviations are used in this manuscript:

RGC	Retinal ganglion cells
mtDNA	Mitochondrial DNA
ROS	Reactive oxygen species
OS	Oxidative stress
SDU	Standard drinking units
BCVA	Best-corrected visual acuity
IOP	Intraocular pressure
AL	Axial length
OCT	Optical coherence tomography
RNFL	Retinal nerve fiber layer
RGC	Retinal ganglion cell layer
PVI	Povidone-iodine
AOPP	Advanced oxidation protein products
LOOH	Lipid hydroperoxides
ELISA	Enzyme-linked immunosorbent assay
IL-1 β	Interleukin-1 β
IFN- γ	Interferon-γ
TNF-α	Tumor necrosis factor α
TAS	Total antioxidant status
GPX	Glutathione peroxidase
GRD	Glutathione reductase
SOD	Superoxide dismutase
G6PDH	Glucose 6 Phosphate Dehydrogenase
SOD3	Extracellular superoxide dismutase
cGMP	Cyclic guanosine monophosphate
IL-1ra	Interleukin 1 receptor antagonist
CHAID	Chi-squared Automatic Interaction Detection
OX/AntiOX	Oxidant/antioxidant
OCR	Overall classification rate
AUC	Area under curve
TOS	Total oxidant status
RNS	Reactive nitrogen species
