# Supplementary material for: Oxidative Stress and Inflammatory Biomarkers in Aqueous Humor and Blood of Patients with Leber’s Hereditary Optic Neuropathy
Source: Antioxidants (Basel). 2025 Dec 30;15(1):51. doi: 10.3390/antiox15010051 (PMC12838384; doi:10.3390/antiox15010051)
Supplement: Supplementary file 1 [file antioxidants-15-00051-s001.zip › antioxidants-4045211-supplementary.pdf]

antioxidants

MDPI

Supplementary Table S1. Oxidative stress and inflammatory biomarkers.

Markers	Full name	Category	Biological meaning	Clinical relevance
AOPP	Advanced Oxidation Protein Products	Oxidative stress	Products generated by irreversible oxidative modification of plasma proteins	Marker of chronic protein oxidative damage and systemic oxidative stress
LOOH	Lipid Hydroperoxides	Oxidative stress	Primary products of lipid peroxidation in cell membranes	Early indicator of membrane oxidative damage and mitochondrial dysfunction
Nitrotyrosine	Nitrotyrosine	Oxidative/nitrosative stress	Protein modification mediated by reactive nitrogen species	Reflects nitric oxide–related oxidative damage
TAS	Total Antioxidant Status	Antioxidant capacity	Global ability of biological fluids to neutralize free radicals	Lower values indicate reduced antioxidant defense
G6PDH	Glucose-6-phosphate dehydrogenase	Antioxidant support enzyme	Provides NADPH required for antioxidant systems	Alterations indicate impaired redox homeostasis
GPx	Glutathione Peroxidase	Antioxidant enzyme	Enzyme that detoxifies hydrogen peroxide and lipid peroxides	Reflects compensatory antioxidant response
GRD	Glutathione Reductase	Antioxidant enzyme	Regenerates reduced glutathione from its oxidized form	Essential for maintaining intracellular redox balance
SOD / SOD3	Superoxide Dismutase (intra-/extracellular)	Antioxidant enzyme	Converts superoxide radicals into less toxic molecules	First-line defense against oxidative stress
cGMP	Cyclic guanosine monophosphate	Nitric Oxide signaling mediator	Second messenger of nitric oxide pathway	Indicates nitric oxide pathway activity and vascular function
IL-1 ß	Interleukin-1 beta	Pro-inflammatory cytokine	Key mediator of inflammatory response	Elevated levels indicate active inflammation
IL-6	Interleukin-6	Pro-inflammatory cytokine	Mediator of acute and chronic inflammation	Marker of inflammatory activation
IL-12p40	Interleukin-12 subunit p40	Pro-inflammatory cytokine	Subunit shared by IL-12 and IL-23 involved in Th1 immune responses	Reflects activation of cell-mediated inflammatory pathways
Leptin	Leptin	Metabolic / inflammatory mediator	Hormone with immunomodulatory and neuroprotective roles	Altered levels may be linked to optic nerve vulnerability

https://doi.org/10.3390/antiox15010051

IFN- γ	Interferon gamma	Pro-inflammatory cytokine	Cytokine produced by T cells and NK cells that promotes macrophage activation	Associated with neuroinflammation and immune-mediated tissue damage
TNF-α	Tumor Necrosis Factor alpha	Pro-inflammatory cytokine	Cytokine involved in inflammation and apoptosis	Associated with neurodegeneration and tissue damage
IL-1ra	Interleukin-1 receptor antagonist	Anti-inflammatory cytokine	Endogenous inhibitor of IL-1 signaling	Reflects compensatory anti-inflammatory response
IL-4	Interleukin-4	Anti-inflammatory cytokine	Promotes Th2 immune response and suppresses pro-inflammatory signaling	Protective role in controlling inflammation
IL-10	Interleukin-10	Anti-inflammatory cytokine	Suppresses pro-inflammatory pathways	Reduced levels may favor inflammatory imbalance
IL-13	Interleukin-13	Anti-inflammatory cytokine	Regulates immune response and tissue remodeling	Reduced levels may be associated with chronic inflammation

Description and clinical relevance of the oxidative stress and inflammatory biomarkers measured in the study.

https://doi.org/10.3390/antiox15010051
